# Effects of Green Tea Gargling on the Prevention of Influenza Infection in High School Students: A Randomized Controlled Study

**DOI:** 10.1371/journal.pone.0096373

**Published:** 2014-05-16

**Authors:** Kazuki Ide, Hiroshi Yamada, Kumi Matsushita, Miki Ito, Kei Nojiri, Kiichiro Toyoizumi, Keiji Matsumoto, Yoichi Sameshima

**Affiliations:** 1 Department of Drug Evaluation and Informatics, Graduate School of Pharmaceutical Sciences, University of Shizuoka, Shizuoka, Japan; 2 Department of Pharmacy, Kikugawa General Hospital, Shizuoka, Japan; 3 Department of Internal Medicine, Omaezaki Municipal Hospital, Shizuoka, Japan; University of Ottawa, Canada

## Abstract

**Background:**

The anti-influenza virus activity of green tea catechins has been demonstrated in experimental studies, but clinical evidence has been inconclusive. School-aged children play an important role in the infection and spread of influenza in the form of school-based outbreaks. Preventing influenza infection among students is essential for reducing the frequency of epidemics and pandemics. As a non-pharmaceutical intervention against infection, gargling is also commonly performed in Asian countries but has not yet been extensively studied.

**Methods and Findings:**

A randomized, open label, 2-group parallel study of 757 high school students (15 to 17 years of age) was conducted for 90 days during the influenza epidemic season from December 1st, 2011 to February 28th, 2012, in 6 high schools in Shizuoka Prefecture, Japan. The green tea gargling group gargled 3 times a day with bottled green tea, and the water gargling group did the same with tap water. The water group was restricted from gargling with green tea. The primary outcome measure was the incidence of laboratory-confirmed influenza using immunochromatographic assay for antigen detection. 757 participants were enrolled and 747 participants completed the study (384 in the green tea group and 363 in the water group). Multivariate logistic regression indicated no significant difference in the incidence of laboratory-confirmed influenza between the green tea group (19 participants; 4.9%) and the water group (25 participants; 6.9%) (adjusted OR, 0.69; 95%CI, 0.37 to 1.28; *P* = 0.24). The main limitation of the study is the adherence rate among high school students was lower than expected.

**Conclusions:**

Among high school students, gargling with green tea three times a day was not significantly more efficacious than gargling with water for the prevention of influenza infection. In order to adequately assess the effectiveness of such gargling, additional large-scale randomized studies are needed.

**Trial Registration:**

ClinicalTrials.gov NCT01225770

## Introduction

Influenza epidemics are a perennial public health problem worldwide. Influenza causes acute respiratory illness, and can lead to severe complications with mortality risk, such as pneumonia and encephalitis [1], [2]. Infection in schools is especially problematic, as close interaction among students allows the virus to be easily transmitted among them, and subsequently to their families and communities [3]–[6]. Therefore, prevention is an essential public health measure. The main strategy for preventing influenza infection is vaccination, but its efficacy and effectiveness depend on the strain of the virus [7], and it has the drawback of limited supply [8], [9]. Neuraminidase inhibitors are also used for prevention, but several reports have shown that they have a limited effect, and that viral resistance to inhibitors such as oseltamivir has been gradually increasing [10]–[14]. For these reasons, a variety of non-pharmaceutical public health interventions to reduce morbidity have been suggested, including facemasks, hand hygiene, and gargling [15]–[17]. In Asian countries, and in Japan especially, gargling is recommended and commonly performed [18]. Gargling has not been extensively studied, and there have been few registered outcome studies of its relation to influenza infection. However, one randomized trial examining upper respiratory illness found that gargling with water reduced the rate of infection by 36% compared with a non-gargling control group [19].

While a variety of non-pharmaceutical public health interventions have been suggested, they do not appear to have had a substantial effect on influenza infection rates. Infection rates have remained consistently high, with the peak percentages of total outpatient visits to U.S. healthcare providers for influenza-like illness being 7.7% during the H1N1 pandemic season in 2009, and 4.5% in the most recently reported 2010-11 season, according to the Outpatient Influenza-like Illness Surveillance Network [20]. In terms of school-related influenza infections, at one New York City high school during the 2009 pandemic season, the rate of infection among students was 3.5 times higher than among the school’s staff [21], indicating potential infection routes related to hygiene and interaction patterns among students. Thus, improved public health interventions performed by students may help to prevent epidemics and pandemics related to school-based outbreaks.

The present study focused on a novel non-pharmaceutical public health intervention against influenza epidemics in schools: gargling with green tea. Green tea is one of the most widely consumed beverages in the world, and its chemical components such as catechins and theanine have a variety of health benefits [22], [23]. Experimental studies have shown that green tea catechins have several anti-influenza virus activities *in vitro*. Regarding infectivity, the highly-bioactive catechin (-)-epigallocatechin gallate has been reported to inhibit plaque formation, adsorption, and hemmagglutination by influenza A and B viruses in Madin-Darby canine kidney cells [24], [25]. In terms of clinical studies, a number of small-scale preliminary studies on the use of tea components for influenza infection prevention have previously been reported. One study suggested that gargling with black tea extracts had a preventive effect on influenza infection over a 5-month period; however, the diagnostic criterion used in that study was based on hemagglutinine antibody titer level, and the control group did not gargle [26]. Our group has also carried out clinical studies on the use of green tea components for the prevention of influenza infection. One was a small prospective cohort study on the effectiveness of gargling with tea catechin extracts among 124 elderly nursing home residents [27]. It found that such gargling significantly reduced the rate of influenza infection. Another study was a randomized controlled trial of 200 health care workers which investigated the effect that capsules of catechins and theanine from green tea had on clinically-defined influenza [28]. That study found that the rate of influenza infection was approximately three times lower in the catechin/theanine group than in the placebo group.

Based on this background, we conducted a randomized, open label, 2-group parallel study to evaluate the clinical efficacy of green tea gargling on the prevention of influenza infection among high school students in Japan.

## Methods

### Design Overview

A randomized, open label, 2-group parallel study was conducted to compare the efficacy of green tea gargling with water gargling for 90 days during the influenza epidemic season, from 1 December 2011 to 28 February 2012. The protocol for this study and supporting CONSORT checklist are available as supporting information; see Checklist S1 and Protocol S1.

### Setting and Participants

We recruited a total of 2,838 high school students (15 to 17 years of age) who attended 6 high schools in the Kakegawa and Ogasa districts of Shizuoka Prefecture, Japan. Recruitment was performed at these schools by posters and announcements at school assemblies. Participants were excluded according to the following criteria: tea allergy (history of asthma, skin rashes, and other indications); history of influenza infection within the previous 6 months; and immune disease or severe cardiac, respiratory, renal, or hepatic dysfunction diagnosed by a medical doctor.

The participants completed a self-administered questionnaire to assess baseline characteristics including age, sex, body mass index (BMI), vaccination for influenza virus, use of public transportation, and type of school club (sports- or culture-related). Green tea consumption habits (defined as≥200 mL/day) were also recorded.

### Randomization and Interventions

Eligible participants were randomized by a computer generated permuted block randomized schema, and stratified according to school and class, at the Data Management Center of Shizuoka General Hospital in Japan. Participants in the green tea gargling group were provided each day with bottled green tea (500 mL) containing a catechin concentration of 37±0.2 mg/dL, including approximately 18% (-)-epigallocatechin gallate manufactured by the Kakegawa Tea Merchants Association. Participants in the water gargling group were asked to gargle with tap water, and were asked not to gargle with green tea during the study. Consumption of green tea and other tea was not restricted for either group. The concentration of catechins in the tea was measured by high-performance liquid chromatography based on the average concentration in 10 bottles from the same production lot (September 2011) used for gargling in the study.

The participants were asked to gargle at least 3 times a day (after arriving at school, after lunch, and after school). They were also asked to complete a questionnaire each day concerning the occurrence of influenza infection, preventive measures (hand washing and facemasks), any adverse events, and their daily adherence to the gargling regimen. The questionnaires were collected every 2 weeks by the students’ homeroom teachers, and safety monitoring was carried out carefully throughout the study. Data management and safety monitoring were assisted by the high schools’ vice principals and head teachers.

All participants and their guardians gave written informed consent before entering the study. The study protocol was approved by the ethics committee of the University of Shizuoka and was conducted in accordance with the Declaration of Helsinki.

### Outcomes and Follow-up

The primary outcome measure was the incidence of laboratory-confirmed influenza infection with viral antigen detected by immunochromatographic assay. This test was performed when an influenza-like symptom occurred. Nasal swab samples for this assay were collected by medical doctors. The sensitivity of rapid influenza diagnostic tests including the immunochromatographic assay was approximately 64.4% (59.0–70.1%) for type A virus, and 52.2% (45.0–59.3%) for type B virus [29]. Two secondary outcome measures were also examined. The first was the incidence of clinically defined influenza infection, specified as fever(≥37.8 °C) plus any 2 of the following additional symptoms: cough, sore throat, headache, or myalgia [30]. The other secondary outcome was the time for which the patient was free from clinically defined influenza infection, i.e., the period between the start of the intervention and the first diagnosis of infection.

### Statistical Analysis

Based on our previous studies, we estimated that laboratory-confirmed influenza infection (primary outcome) would occur in 4% of participants in the green tea gargling group and 10% of those in the water gargling group. The sample size was estimated as 306 for each group at a power level of 0.80 and a 2-sided α level of 0.05. Estimating a 15% drop out rate, we set the total sample size at 720.

The full analysis set and per protocol set were used for all efficacy analysis, and safety analysis was performed for the full analysis set. An interim analysis was not planned. The full analysis set was determined by excluding participants from the intention-to-treat population according to the following criteria: no gargling carried out, and/or no gargling data collected, and/or withdrawal from the study and refusal to have data included in the study. In addition to these criteria, the per-protocol set was defined according to the following criteria: adherence rate of gargling at or above 75%, and absence of green tea gargling when in the water gargling group.

A chi-square test was performed for categorical comparisons of the data. Differences in the mean values of continuous measurements were tested by Student’s *t*-test or Mann-Whitney U test. Multivariate logistic regression analysis was used to provide adjusted odds ratio (OR) estimates and a 95% confidence interval (CI) for the association between green tea gargling and the incidence of influenza infection. Cumulative incidence rates were determined by Kaplan-Meier method. The Cox proportional hazards regression model was used to evaluate the association between green tea gargling and the time for which the participants were free from clinically defined influenza infection, and was adjusted for potential confounding variables after confirmation of the population hazard assumption. The participants that were free of influenza infection for a period of 90 days were censored at this time. Baseline characteristics with *P*<0.20 were considered as potential confounding variables. They were defined by multivariate logistic regression analysis and transferred to the Cox proportional hazard model. In these multivariate analyses, vaccination was also considered an independent variable because of its medical implications.

*P*<0.05 was considered statistically significant. All statistical analyses were performed using SPSS for Windows, version 21.0 (IBM Corp., Armonk, NY).

## Results

**Figure 1:**
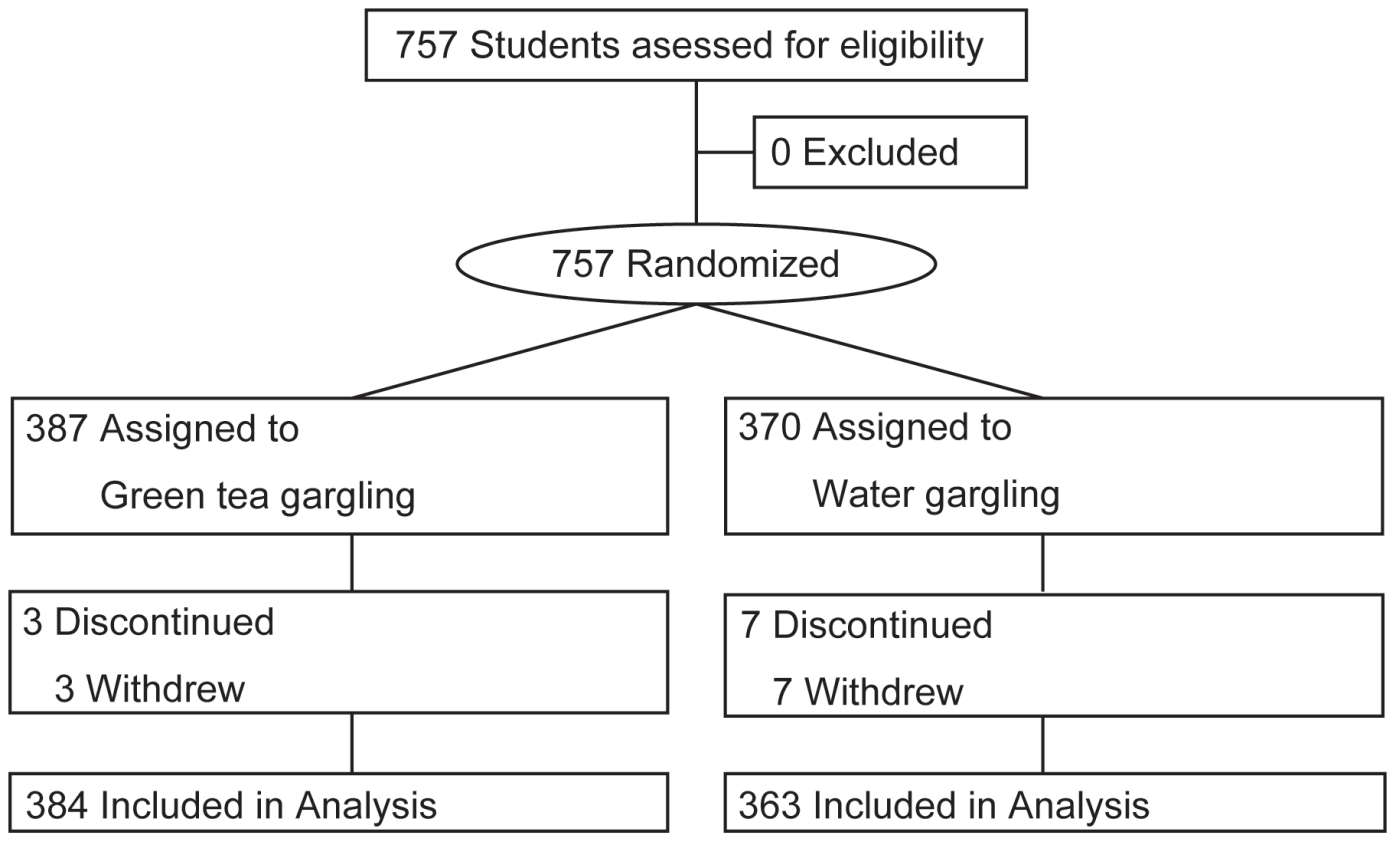
Flow diagram for study.

Among the 6 high schools in the Kakegawa and Ogasa districts, 757 of 2,838 students gave written informed consent and were assessed for eligibility; none of the students were excluded according to the exclusion criteria. All of the 757 participants were enrolled and randomly assigned to an intervention; 387 were allocated to the green tea gargling group, and 370 to the water gargling group. Between assignment and intervention, 3 participants in the green tea group and 7 in the water group withdrew from the study. All 10 participants who withdrew did so because of their refusal to give their consent. 747 participants completed the study (Figure 1). According to the per-protocol set criteria, the gargling adherence rate was 73.7% in the green tea group and 67.2% in the water group.

### Baseline Characteristics

The baseline characteristics of the participants in the full analysis set (n = 747: green tea group, n = 384; water group, n = 363) and per protocol set (n = 527: green tea group, n = 283; water group, n = 244) are shown in Table 1. The mean age of the participants was 16.1 years, and the age range was 15 to 17 years. Baseline characteristics were well balanced, except for type of school club (sport- or culture-related) (*P* = 0.06 in the full analysis set; *P* = 0.17 in the per protocol set). Therefore, type of school club was considered a confounding variable in the multivariate analyses. In addition, the influenza vaccination for use in the 2011-12 season contained the following three vaccine viruses: an A/California/7/2009 (H1N1) pdm9-like virus, an A/Perth/16/2009 (H3N2)-like virus, and a B/Brisbane/60/2008-like virus, and the subtype of influenza A virus circulating in Japan at that time was H3N2. The vaccination was administered from October until mid-November, 2011.

**Table 1:** Baseline characteristics of study participants.

Full analysis set	Green tea gargling	Water gargling	
Characteristics	(n = 384)	(n = 363)	*P* value
Age, mean (SD) [range]	16.1 (0.7) [15]–[17]	16.2 (0.7) [15]–[17]	0.44†
Sex, No. (%)			
Men	224 (58.3)	199 (54.8)	0.33§
Women	160 (41.7)	164 (45.2)	
BMI, mean (SD) [range]	20.5 (2.7) [15.4–36.7]	20.6 (2.4) [15.1–34.9]	0.23‡
Vaccination for influenza virus, No. (%)	139 (36.2)	126 (34.7)	0.67§
Hand washing††, No. (%)	312 (81.3)	303 (83.5)	0.43§
Facemasks††, No. (%)	52 (13.5)	52 (14.3)	0.76§
Type of clubs, No. (%)			
Sports	253 (65.9)	214 (58.9)	0.06§
Culture	131 (34.1)	149 (41.1)	
Public transportation, No. (%)	181 (47.1)	157 (43.3)	0.29§
Green tea drinking habit–∥, No. (%)	270 (70.3)	261 (71.9)	0.63§
Per protocol set	Green tea gargling	Water gargling	
Characteristics	(n = 283)	(n = 244)	*P* value
Age, mean (SD) [range]	16.1 (0.7) [15]–[17]	16.1 (0.7) [15]–[17]	0.72†
Sex, No. (%)			
Men	163 (57.6)	130 (53.3)	0.32§
Women	120 (42.4)	114 (46.7)	
BMI, mean (SD) [range]	20.5 (2.7) [15.4–36.7]	20.6 (2.4) [16.0–34.9]	0.37‡
Vaccination for influenza, No. (%)	109 (38.5)	88 (36.1)	0.56§
Hand washing††, No. (%)	243 (85.9)	217 (89.0)	0.29§
Facemask††, No. (%)	41 (14.5)	30 (12.3)	0.46§
Type of club, No. (%)			
Sports	179 (63.3)	140 (57.4)	0.17§
Culture	104 (36.7)	104 (42.6)	
Public transportation, No. (%)	134 (47.3)	115 (47.1)	0.96§
Green tea drinking habit–∥, No. (%)	208 (73.5)	178 (73.0)	0.89§

Abbreviation: BMI, body mass index.

† *P* value based on Student’s *t*-test.

‡ *P* value based on Mann-Whitney U test.

§ P value based on chi-square test.

†† Hand washing and facemasks performed 4 days per week or more during study period.

–∥ Green tea drinking habit defined as drinking over 200 mL (one cup) of green tea per day.

### Laboratory Confirmed Influenza Infection and Other Outcomes

During the study, laboratory confirmed influenza infection occurred in a total of 44 participants (5.9%) in the full analysis set, and 36 (6.8%) in the per protocol set participants were infected. Among the 44 confirmed participants in the full analysis set, 42 had type A antigen, and 2 had type B. Clinically defined influenza was diagnosed in 113 participants (14.9%) in the full analysis set and 84 (15.9%) in the per protocol set. No participants had more than one influenza infection during the observation period.

Multivariate logistic regression analysis showed that there was no difference between the green tea and water groups in the incidence of laboratory-confirmed influenza infection (Full analysis set: Adjusted OR, 0.69; 95% CI 0.37 to 1.28; *P* = 0.24. Per protocol set: Adjusted OR, 0.86; 95% CI 0.44 to 1.69; *P* = 0.66) (Table 2). In addition, there was no significant difference in the incidence of clinically defined influenza in the two groups in the full analysis set (52 participants (13.5%) in the green tea group, and 61 participants (16.8%) in the water group; Adjusted OR, 0.75; 95% CI 0.50 to 1.13; *P* = 0.17). In the per protocol set, the incidence of clinically defined influenza was lower in the green tea group (39 participants, 13.8%) than in the water group (45 participants, 18.4%); however, this difference was not significant (adjusted OR, 0.69; 95% CI 0.43 to 1.11; *P* = 0.13) (Table 2).

**Table 2:** Results of multivariate logistic regression analysis for influenza infection.

Full analysis set				
	Laboratory confirmed influenza||		Clinically defined influenza¶	
Variable	OR (95% CI)	*P* value	OR (95% CI)	*P* value
Allocation				
Green tea	0.69 (0.37 to 1.28)	0.24	0.75 (0.50 to 1.13)	0.17
Water	1 [Reference]		1 [Reference]	
Vaccination	1.06 (0.56 to 2.00)	0.85	1.02 (0.67 to 1.55)	0.94
Club (sport related)	1.35 (0.70 to 2.59)	0.38	1.51 (0.98 to 2.33)	0.07
Per protocol set				
	Laboratory confirmed influenza		Clinically defined influenza	
Variable	OR (95% CI)	*P* value	OR (95% CI)	*P* value
Allocation				
Green tea	0.84 (0.44 to 1.69)	0.66	0.69 (0.43 to 1.11)	0.13
Water	1 [Reference]		1 [Reference]	
Vaccination	0.73 (0.35 to 1.51)	0.39	0.84 (0.51 to 1.38)	0.49
Club (sport related)	1.02 (0.51 to 2.04)	0.97	1.48 (0.90 to 2.43)	0.13

|| Laboratory confirmed influenza with viral antigen detected by immunochromatographic assay.

¶ Clinically defined influenza infection diagnosed as fever(≥37.8 °C) plus any 2 of following symptoms: cough, sore throat, headache, or myalgia.

**Figure 2:**
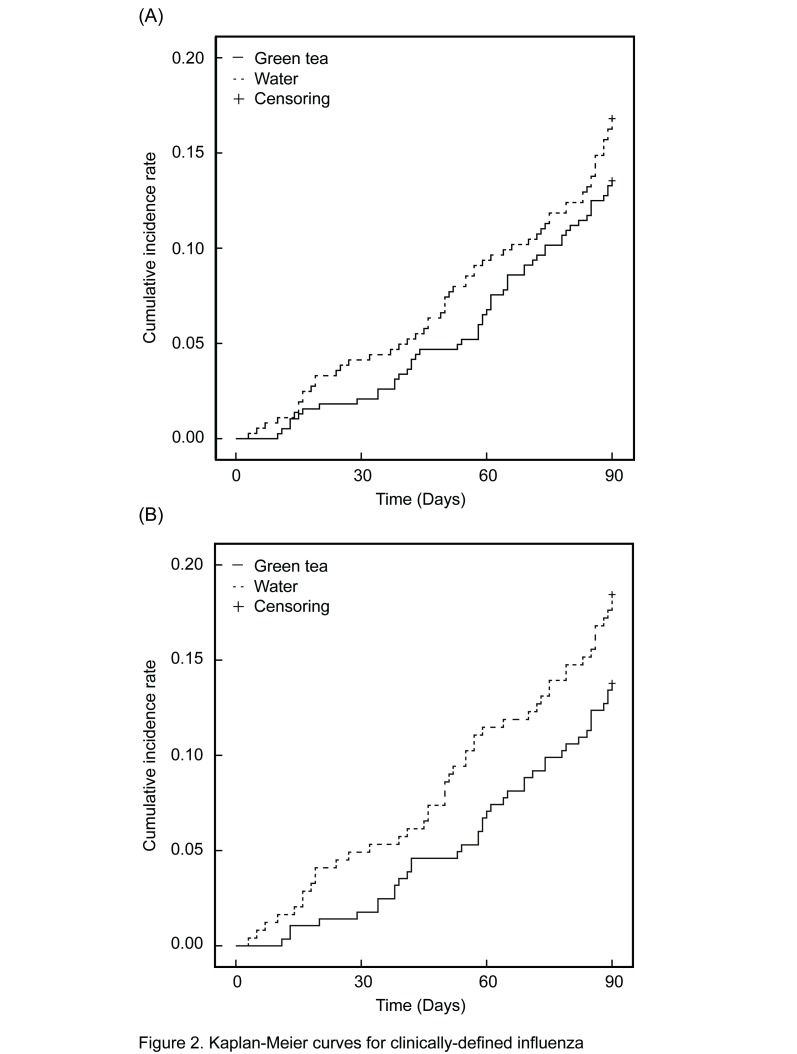
Kaplan-Meier curves for clinically-defined influenza. (A) Full analysis set, (B) Per protocol set.

Kaplan-Meier curves are shown in Figure 2. The time before diagnosis of clinically defined influenza infection was estimated with the Cox proportional hazard regression model. There was no difference between the two groups (Full analysis set: Adjusted HR, 0.77; 95% CI 0.53 to 1.11; *P* = 0.16. Per protocol set: Adjusted HR, 0.71; 95% CI 0.46 to 1.08; *P* = 0.11) (Table 3).

**Table 3:** Results of Cox proportional hazards regression model for clinically defined influenza infection.

Full analysis set		
Variable	HR (95% CI)	*P* value
Allocation		
Green tea	0.77 (0.53 to 1.11)	0.16
Water	1 [Reference]	
Vaccination	1.00 (0.69 to 1.48)	0.97
Club (sport related)	1.46 (0.98 to 2.20)	0.06
Per protocol set		
Variable	HR (95% CI)	*P* value
Allocation		
Green tea	0.71 (0.46 to 1.08)	0.11
Water	1 [Reference]	
Vaccination	0.84 (0.54 to 1.33)	0.46
Club (sport related)	1.42 (0.91 to 2.29)	0.12

Abbreviation: HR, hazard ratio; CI, confidence interval.

No adverse events were observed in the participants of either group during the study.

## Discussion

This randomized clinical study was conducted to compare the efficacy of green tea gargling with water gargling in preventing influenza infection among high school students in Japan. While several previous experimental and clinical studies had suggested that tea components, especially catechins, were effective for preventing influenza infection, this study of a 90-day green tea gargling intervention among high school students did not show a significant difference compared with water gargling.

It should be pointed out, however, that there are several limitations to this study. The main limitation was adherence rate. In the sample size estimation, the expected dropout rate was set to 15%, but in practice the non-adherence rate in the full analysis set was much higher at 29%. The reduction in influenza infection was greater when the analysis were restricted to the per protocol set; therefore, if the adherence rate could be improved in future studies, it may be possible to detect a significant difference between the water and green tea gargling groups. A related issue is practical methods of ensuring a high adherence rate. In this study, it was difficult to maintain such a rate among the high school students. It is possible that they forgot or found the protocol troublesome. In future studies, the adherence rate might be improved by interventions such as sending reminders to participants. Moreover, in future studies it may be possible to detect laboratory-confirmed influenza infection more accurately by using more sensitive diagnostic tools.

The non-blinded design of this study may also be a limitation. With this type of design, there is the potential for placebo effects. However, it is difficult to make a placebo green tea beverage because of the unique taste of green tea. That being said, in future studies it may be appropriate to attempt to use such a beverage in the control group to clearly reveal the effects of green tea gargling. Doing so may also improve the adherence rate of the gargling control group. In this study, the adherence rate of the water gargling group (67.2%) was lower than that of the green tea gargling group (73.7%). This may be related to differences in sustaining motivation due to differences in the participants’ perception of the effectiveness of their treatments. Therefore, the intervention conditions for the two groups should be made as similar as possible.

An additional possible limitation is the living environment of the participants. Compared with our previous study of elderly nursing home residents with a mean age of over 80 years [27], the young participants in this study had many more opportunities to be infected with influenza because of their close interaction with each other. This observation is supported by studies showing the impact of school closures on reducing the severity of influenza epidemics and pandemics, including excess death rate [3]. In addition, the students were involved in a variety of daily activities that carried a risk of infection, such as the use of public transportation, as well as movement through other crowded places.

Gargling frequency may also have been a limitation. The frequency was set at 3 times a day (after arriving at school, after lunch, and after school), based on our previous study of green tea gargling among elderly nursing home residents. However, this frequency may not have been high enough to have an effect on high school students, who interact with others at a much higher rate than nursing home residents, and therefore have many opportunities to become infected. It is possible that more detailed determination of the most effective frequency and duration of gargling in each particular population and site may influence the rate of prevention of infection.

Baseline green tea consumption is also a potential limitation of this study. Over 70% of the participants in both groups were regular green tea drinkers, consuming 1 cup (200 mL) or more of the beverage per day. As our previous observational study of elementary school students indicated that consumption of 3 to 5 cups (600 to 1000 mL) of green tea per day was associated with a significantly reduced incidence of influenza infection (adjusted OR of 0.62 compared with<1 cup/day) [31], this baseline green tea consumption rate may have influenced the outcome of the present study. In future studies, the quantity of green tea consumed daily by participants should be recorded to control for the confounding effect of green tea consumption.

In addition, as a high rate of clinically-defined influenza infection was found in this study, it may be fruitful in future studies to examine the effect of gargling with water or green tea in combination with other personal hygiene practices and health behaviors, such as hand hygiene with ethanol. For example, a previous clinical study on the combined effects of hand hygiene and facemasks on infection prevention found that, while the use of facemasks alone was not efficacious in comparison with a non-interventional control, the combination of facemasks with hand hygiene significantly reduced the infection rate [16].

In summary, this study reveals that, in order to adequately assess the effects of green tea gargling on the prevention of influenza infection, additional large-scale randomized studies with sufficiently high adherence rates and maximally effective gargling methods are needed.

## Supporting Information

Checklist S1 **CONSORT checklist.**
(DOC)

Protocol S1 **Study protocol.**
(PDF)
