# Supplementary material for: Effects of Green Tea Gargling on the Prevention of Influenza Infection in High School Students: A Randomized Controlled Study
Source: PLoS One. 2014 May 16;9(5):e96373. doi: 10.1371/journal.pone.0096373 (PMC4023996; doi:10.1371/journal.pone.0096373)
Supplement: Protocol S1 — Study protocol. (PDF) [file pone.0096373.s002.pdf]

Study protocol (translated in English)

Trial Registration: Clinical Trials.gov ID: NCT01225770

Title:

Effects of Green Tea Gargling on the Prevention of Influenza Infection in High School Students: A Randomized Controlled Study

Ethics Committee:

22-16 (2010/9/6)
Ethics committee at the University of Shizuoka

Sponsors and Collaborators: University of Shizuoka
Information provided by: University of Shizuoka

Brief summary:

Experimental and clinical studies in adults have reported that green tea catechins prevent influenza infection. In this clinical randomized study, we aimed to determine the effects of gargling with green tea on the prophylaxis of influenza infection among high school teenagers.

Purpose:

The purpose of this study is to evaluate the effects of gargling with green tea on preventing influenza infection among high school students.

Background;

Influenza infection is the principal cause of acute respiratory illnesses and occurs in epidemic or pandemic worldwide. It spreads easily among high school students and from them to the rest of the community; therefore the prevention of influenza is very important.

Experimental studies have revealed that some components of green tea can prevent influenza infection *in vitro*. In our previous clinical studies, we showed that gargling with green tea catechins had possibilities to preventing influenza infection in adults; however, little clinical evidence in teenagers has so far been existed. Based on this background, we designed a randomized study to evaluate the clinical efficacy of gargling with green tea in preventing influenza infection among high school students.

Study Type: Interventional
Study Design:

Prevention, Randomized, Open label, Parallel Assignment, Safety/Efficacy Study

Expected Total Enrollment: 720
Study start: December 2010; Study completion: March 2012

Primary Outcomes:

- the incidence rates of influenza infection, until 3 months gargling

Secondary Outcomes:

- severity and duration of influenza symptoms
- influenza-free time during the study period
- incidence of complications or hospitalization and days of absence from school

- occurrence of the adverse events

Arm Type:
  Comparator, water
    Gargling with water three times daily for 90 days
  Experimental, green tea
    Gargling with green tea three times daily for 90 days

Eligibility Criteria:

Inclusion Criteria:

- aged from 15 to 20 years
- obtained written informed consent from both the student and parent before participation
- possible to gargle during 90 days
- possible to fill in questionnaire

Exclusion Criteria:

- Possessing allergy to green tea
- Possessing history of influenza infection within 6 months before entering the study
- Possessing severe immune disease or whole body infection
- severe cardiac, respiratory, renal, or hepatic dysfunction
- diagnosed as inadequate to participate in the study by student doctor

Location Information:
  Study chairs or principal investigators:
Hiroshi Yamada, MD, PhD, Principal Investigator, University of Shizuoka
Professor
Department of Drug Evaluation & Informatics
Graduate School of Pharmaceutical Sciences
University of Shizuoka
52-1 Yada, Suruga-ku, Shizuoka, 422-8526
Japan
TEL & FAX: +81-54-264-5762
e-mail: hyamada@u-shizuoka-ken.ac.jp

Health Authority: Ministry of Health, Labor and Welfare, Japan

<u>Original study protocol (in Japanese)</u>

臨床試験実施計画書

2010 年 7 月 1 日作成

タイトル：
高校生におけるインフルエンザ予防のための緑茶うがい効果の検証：ランダム化比較試験

1. 背景と目的

インフルエンザは冬季に流行する急性の上気道感染症である。インフルエンザは高齢者や小児、免疫能低下者において、肺炎、脳炎など重篤な合併症を引き起こすことがあり、その予防･治療対策は国民の健康･福祉の向上のために必須である。特に、集団生活が行わる若年者の通う学校での予防対策は、2009 年に生じた新型インフルエンザ流行時の罹患者に若年者が多ことも相俟って、重要な課題となっている。

緑茶に多く含まれるポリフェノールの一種である茶カテキンは、抗酸化作用をはじめ、抗菌･抗ウイルス作用など様々な生理活性を有し、インフルエンザウイルスに対する感染予防効果が基礎的研究により報告されている。一方、ヒトにおける効果を科学的に検討した報告はわずかであり、臨床的な評価も未だ定まっていない。我々はこれまでに、高齢者を対象とした前向きコホート研究並びに健常成人を対象としたランダム化二重盲検比較試験により、茶カテキンを用いたうがいがインフルエンザ予防に有効である可能性を示した。また、小学生を対象とした疫学調査において、適量の緑茶摂取とインフルエンザの発症との間に負の関連があることを報告した。そこで今回、高校生を対象とするランダム化比較試験を計画し、インフルエンザ予防のための緑茶うがいの効果を検証することとした。

2. 対象

以下の選択・除外基準を満たす高校生ボランティア

2.1. 選択基準

- 研究参加前に、被験者本人及び保護者から文書同意が得られていること
- 年齢:15 歳以上 20 歳未満、ただし性別は問わない
- 90 日間のうがいが可能なこと
- アンケート（調査票）の記入が可能なこと

2.2. 除外基準

- 茶アレルギー（喘息、発疹等の既往）のある者
- 研究開始前 6 ヶ月以内にインフルエンザに罹患した者
- その他医師が本試験の対象として不適当と判断した者

3. 研究の方法

3.1 スケジュール

被験者及び保護者から文書同意を得た後、緑茶うがい群または水うがい群にランダムに割付け、1 日 3 回（登校時、昼休み後、帰宅時）のうがいを 90 日間行う（盲検化は行わない）。被験者に提供される緑茶は成分の統一を図るため、ペットボトルで市販されているものとする。

割付は、静岡県立総合病院に設置したデータマネジメントセンターにおいて、コンピュータによる疑似乱数発生の下で行う。ランダム化の方法は層別ブロックランダム化法を用い、割付調整因子は学校及びクラスとする。

うがい実施期間： 2010 年 11 月～2012 年 3 月上旬（連続 90 日間）

3.2. エンドポイント

以下の項目を緑茶うがい群及び水うがい群の両群間で比較する。

3.2.1. 主要エンドポイント

- 試験期間中のインフルエンザ罹患の有無

3.2.2. 副次エンドポイント

- インフルエンザ罹患時の症状の種類（発熱、倦怠感、筋肉痛、鼻水、咳、痰等）と持続期間
- うがい開始からインフルエンザ発症までの期間
- 合併症（肺炎、脳炎等）罹患、入院、病欠･遅刻･早退の有無
- 試験期間中の有害事象発症の有無

4. 目標症例数

720 例（緑茶うがい群 360 例、水うがい群 360 例）

（症例数設定の根拠: 既に行われた高齢者及び健常成人における茶カテキンのうがいによるインフルエンザ予防効果の検討における結果と実施可能性を踏まえ決定した。）

5. 観察･検査項目

5.1. 罹患状況の調査

インフルエンザに罹患した場合の抗原検査結果、インフルエンザ･感冒に罹患した場合の発症日、症状（発熱、倦怠感、筋肉痛、鼻水、咳、痰等）の有無及び持続期間、合併症（肺炎、脳炎等）・入院の有無及び入院日数、インフルエンザに罹患した場合の病欠･遅刻･早退日数、併用療法（抗インフルエンザ薬の使用（タミフル、リレンザ、その他）等）、学校別･クラス別のインフルエンザ罹患状況、家族のインフルエンザ罹患状況

5.2. 背景因子の調査

学校、学年、クラス、年齢、性別、身長、体重、茶（緑茶、紅茶、ウーロン茶）の飲用習慣（飲用量･期間）、インフルエンザ予防接種状況、試験開始前の健康状態（感冒罹患状況等）

5.3. インフルエンザ罹患の特定

罹患疑い時に受診した医療機関でのインフルエンザ抗原検査が陽性となった場合に、診断確定とする。抗原検査の判定はインフルエンザの診断のための重要項目のため、学校医･養護教員を通じて可能な限り情報収集する。抗原検査の判定が不明の場合は疑い例とする。

6. 倫理的事項

本研究は世界医師会「ヘルシンキ宣言」（2008 年 10 月ソウル改訂）及び厚生労働省「臨床研究に関する倫理指針」（2008 年 7 月改正）に則り、参加するボランティアの人権及び福利を十分に配慮して実施する。

本研究の開始にあたっては、事前に静岡県立大学倫理審査委員会の承認を得る。また、参加するボランティアには、研究担当者より研究内容について十分に説明を行い、理解した上での本人の自由意思並びに保護者の了解による双方からの文書同意が得られてから実施する。

参加するボランティアに対するプライバシーは充分に配慮して、研究を実施する。個人

情報に関しては連結可能な匿名化を行い、個人情報が研究実施高校から外部に漏洩しないよう、細心の注意を払う。

　万が一、有害事象が発生した場合には、最善を尽くして対応する。なお、有害事象が発生した場合の医療行為は全て保険診療内外を含め、金銭での補償は行わない。

7. 参加ボランティアの費用負担

　研究に関わる諸費用は、静岡県立大学 薬学研究院 医薬品情報解析学講座の研究費（厚生労働科研）より補填するため、参加ボランティアの費用負担は発生しない。

8. 研究組織及び役割分担

主任研究者：静岡県立大学 薬学研究院
医薬品情報解析学講座　教授　山田 浩
共同研究者：菊川市立総合病院 薬剤部　松下久美
御前崎市立総合病院 内科　鮫島庸一
静岡県立大学 薬学研究院
医薬品情報解析学講座　井出和希
客員共同研究員　豊泉樹一郎、松本圭司
学部生　伊東未来、野尻　桂

9. 主任研究者等の連絡先

主任研究者：静岡県立大学 薬学研究院
医薬品情報解析学講座　教授　山田 浩
〒422-8526 静岡県静岡市駿河区谷田 52-1
TEL & FAX: 054-264-5762
E-mail: hyamada@u-shizuoka-ken.ac.jp
